# Thorax shape reconstruction from limited CT-digitized palpable landmarks using statistical shape modeling

**DOI:** 10.1007/s11517-026-03624-x

**Published:** 2026-07-11

**Authors:** Ricardo Alcobia, João Folgado, Carlos Quental

**Affiliations:** IDMEC, Instituto Superior Técnico, Universidade de Lisboa, Av. Rovisco Pais, 1, 1049-001 Lisbon, Portugal

**Keywords:** Computational modeling, Statistical shape modeling, Subject-specific, Anatomical landmarks, Skin-to-bone landmark mapping

## Abstract

This study aimed to develop and evaluate algorithms for reconstructing subject-specific thorax geometries from a limited set of skin landmarks, typically collected in motion analysis studies, using statistical shape models (SSMs). A dataset of DICOM CT scans from 76 individuals was used to generate thorax geometries, comprising the sternum, ribs, and vertebrae C6 to T10, and to collect five bone landmarks and their corresponding skin landmarks. Two thorax SSMs were developed from the CT-derived bone geometries, differing in whether skin landmarks were embedded within the SSM. Based on these models, two reconstruction strategies were implemented using an optimization framework: (i) a bone-landmark–based approach in which multiple linear regression was used to map skin landmarks to bone landmarks (SSM-BL), which served as optimization targets, and (ii) a skin-landmark–embedded approach that used skin landmarks directly as optimization targets (SSM-SL). Skin-to-bone mapping accuracy was quantified using mean absolute error (MAE) and root-mean-square error (RMSE). Thorax reconstruction accuracy was assessed using point-to-point surface errors, reported using MAE and RMSE. Skin-to-bone regression models achieved MAE and RMSE below 5 mm for most landmark coordinates. No statistically significant differences were observed between reconstruction strategies. Thorax reconstruction errors ranged from 8.26 ± 1.96 mm to 8.36 ± 1.70 mm (MAE) and from 9.10 ± 1.12 mm to 9.32 ± 1.49 mm (RMSE), depending on the number of retained principal components. The proposed SSM-based methods enable thorax reconstruction from sparse data, capturing global thorax morphology. However, local anatomical details were less accurately represented.

**Graphical Abstract:**

**Figure:**
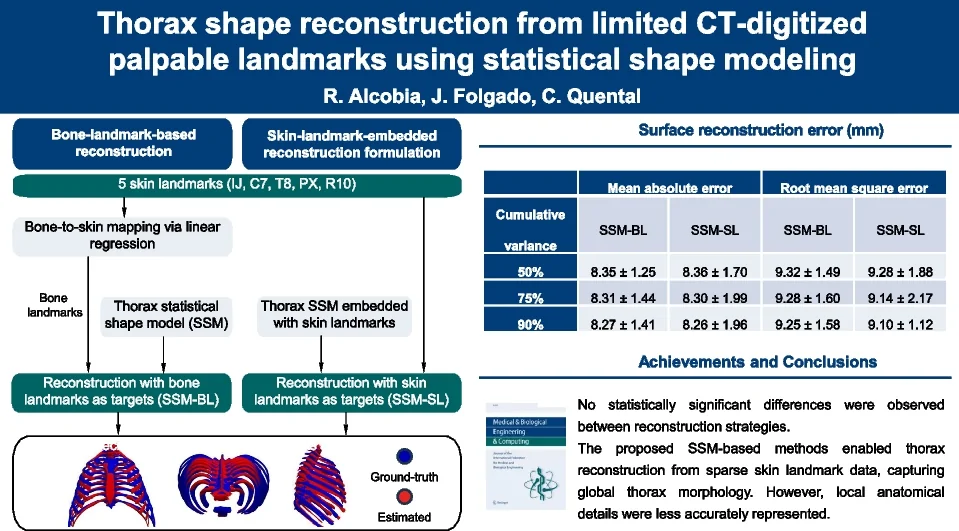

**Supplementary Information:**

The online version contains supplementary material available at https://doi.org/10.1007/s11517-026-03624-x.

## Introduction

Computational biomechanical modeling, including musculoskeletal and finite element approaches, is a powerful tool for understanding the complexities of the human body, providing insights into motion, injury mechanisms, and other applications [1–3]. Despite this potential, translating computational biomechanical models into real-world practice remains challenging. Model development typically involves numerous time-consuming, specialized procedures, which limit accessibility even for experienced professionals. Consequently, novel time- and cost-effective methods for generating subject-specific biomechanical models are essential to enable broader adoption [4].

Building on motion analysis applications, where body landmarks are typically tracked using retroreflective markers placed on the skin, Marques et al. [5] proposed a statistical shape modeling algorithm for reconstructing 3D scapular geometries from a limited set of landmarks. Using skin landmarks and subject-specific variables (sex, age, height, and weight) as input, the method maps skin landmarks onto bone landmarks via linear regression, and uses these bone landmarks as targets in an optimization-based reconstruction algorithm. This approach enabled the reconstruction of scapula shapes, with reported point-to-point surface root-mean-square errors ranging from 1.5 mm to 3.75 mm, supporting its potential for subject-specific computational modeling. Importantly, it opened new avenues for shoulder modeling in scenarios where medical imaging data are unavailable, such as in motion analysis studies.

While statistical shape modeling has proven effective for reconstructing scapular geometry from sparse data, its extension to the entire shoulder complex is challenged by the anatomical interdependence and geometric variability of the involved components. In this context, the thorax serves as a foundational structure in shoulder biomechanics; yet, it remains relatively understudied, likely due to its structural complexity and the interdependent relationships between its components. Research using statistical shape modeling has typically focused either on specific thoracic components or on the thorax as a whole. For example, Clogenson et al. [6] analyzed the shape variability of the second cervical vertebrae using 92 CT scans, Pontiki et al. [7] examined rib curvature and length of thoracic ribs 2–10 in nine subjects, and Wai et al. [8] investigated local variability in thoracic vertebrae T4-T6 using 90 CT scans. Bastir et al. [9], Larsson et al. [10], and Wang et al. [11] created statistical shape models (SSMs) of the thorax using CT scans from 20, 89, and 101 subjects, respectively, to study overall thoracic shape variability. While these studies provide valuable insights into population-level variability and demonstrate the potential of SSMs for 3D morphometric analysis, they largely relied on dense surface meshes or volumetric data obtained from medical image data, and most did not report reconstruction errors to assess their model accuracy. Reconstruction of thorax geometry from a limited set of skin landmarks remains largely unexplored.

The objectives of this study were to develop and evaluate statistical shape modeling algorithms for reconstructing the human thorax, including the sternum, ribs, and vertebrae C6 to T10, from a limited set of skin landmarks typically collected using motion capture systems, and to compare two reconstruction approaches. One approach was based on the method proposed by Marques et al. [5], while the other embedded skin landmarks directly within the SSM, thereby eliminating the need for an intermediate mapping from skin to bone landmarks, which was hypothesized to potentially limit reconstruction accuracy.

## Materials and methods

The methodology of this study involved bone segmentation from CT images and landmark collection from both the segmented bones and CT images. The segmented thorax shapes included the sternum, ribs, and vertebrae C6 to T10. Statistical shape models of the thorax were built both with and without embedded skin landmarks, leading to the development of two reconstruction algorithms from a set of limited skin landmarks. Following the approach presented by Marques et al. [5], one algorithm used linear regression to map skin landmarks onto bone landmarks, which were then used as targets in an optimization problem. The linear regression models mapping skin to bone landmarks received skin landmark coordinates and subject-specific variables (sex, age, height, and weight) as input. The alternative algorithm used a similar optimization approach but used directly the skin landmarks embedded in the SSM as targets. The following sections provide further details on these procedures.

### Dataset description and processing

The dataset used consisted of DICOM CT scans (head/neck/upper extremity protocol) from 76 individuals classified as “White” in the New Mexico Decedent Image Database (NMDID) [12]. None had documented skeletal pathologies. All individuals were adults, evenly divided between females (n = 38) and males (n = 38), with mean ± standard deviation values of 49 ± 17 years for age (range: 20–80 years), 71.6 ± 14.5 kg for weight (range: 41.0–115.0 kg), and 1.71 ± 0.08 m for height (range: 1.52–1.90 m).

Thorax segmentation was performed using TotalSegmentator (v2.11.0) [13], followed by manual verification and refinement in 3D Slicer [14] to ensure anatomical accuracy. The final segmented thorax models consisted of 33 bones, including the sternum, 10 left and 10 right ribs, and vertebrae C6 to T10. This region of interest was selected because it is relevant for studying shoulder biomechanics.

**Fig. 1:**
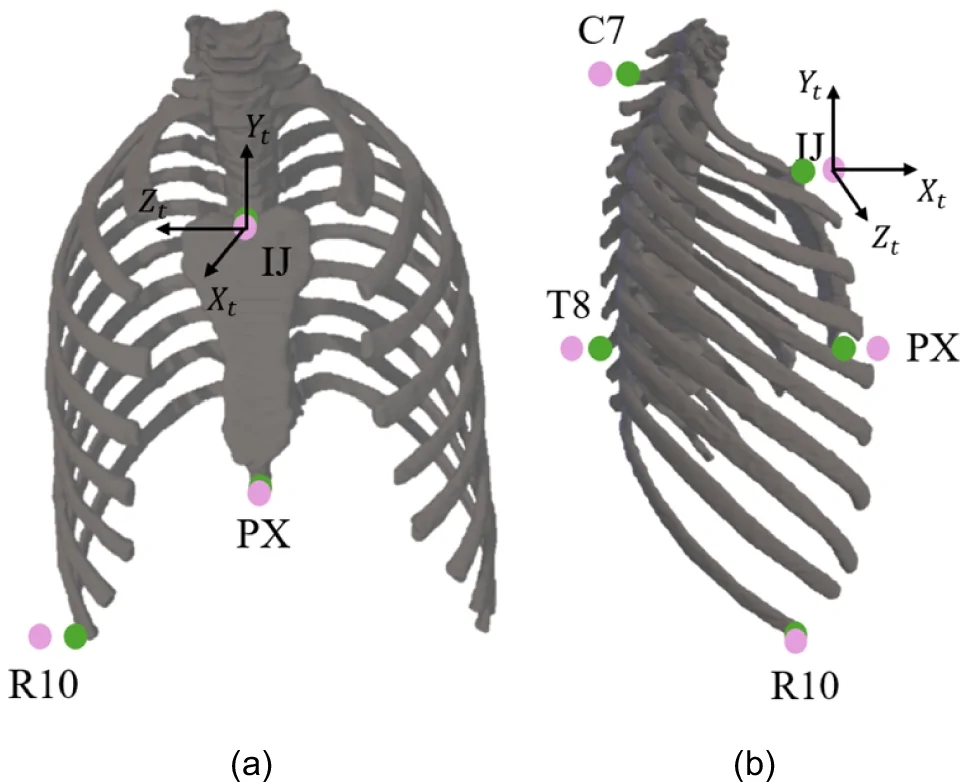
Representative thorax model illustrating the segmented bony anatomy (sternum, ribs, and vertebrae C6–T10), with bone landmarks shown in green and corresponding skin landmarks in pink (C7, T8, IJ, PX, and R10), along with the ISB thoracic reference frame (*X*_t_, *Y*_t_, *Z*_t_) used for landmark standardization

Based on motion analysis studies [15] and following the recommendations of the International Society of Biomechanics (ISB) [16], five bone and skin landmarks were manually collected from the thorax models and corresponding CT data using nmsBuilder [17]. These landmarks included the spinous process of the 7th cervical vertebra (C7), the spinous process of the 8th thoracic vertebra (T8), the *incisura jugularis* (IJ), the *processus xiphoideus* (PX), and the right 10th rib edge (R10) [15]. The R10 landmark, not specified in ISB recommendations, was included to capture variations in thorax width. Skin landmarks were defined as the bone landmarks identified at the skin surface. Their positions were manually located on the skin surface based on the corresponding underlying bone landmarks, simulating typical laboratory procedures in motion analysis. All landmark coordinates were standardized into the ISB thoracic reference frame, enabling inter-subject comparison and further statistical modeling. Figure 1 illustrates a representative thorax model, including the bone and skin landmarks and the associated reference frame.

The dataset was randomly split into training (80%) and test (20%) sets, with the test set reserved for evaluating model performance on unseen data.

### Statistical shape modeling

Two SSMs were constructed from the thorax bone geometries: one composed exclusively of bone surface points (SSM-BL), and another in which the five collected skin landmarks were embedded alongside the bone surface points (SSM-SL). Model construction followed the methodology described by Marques et al. [5].

Briefly, the thorax models were decimated, Laplacian-filtered, and remeshed to obtain 20,000 isotropic vertices per model [18, 19]. To facilitate registration, all models were temporarily normalized by their centroid size (i.e., uniformly scaled in all directions), computed as the square root of the summed squared distances of all vertices from their centroid. Models were then pre-aligned using their principal axes of inertia. A reference model was randomly selected from the SSM training set, after which all other models were rigidly registered to this reference using the Iterative Closest Point (ICP) algorithm implemented in the Open3D Python library [20]. Non-rigid registration was then performed between the reference model and all remaining models to establish point-to-point correspondence using the Bayesian Coherent Point Drift (BCPD) algorithm developed by Hirose [21]. During non-rigid registration, an oversampling factor of three was applied to the target models to maximize the uniqueness of correspondences [22]. When skin landmarks were embedded in the models, explicit correspondence estimation for these landmarks was unnecessary, as their correspondence was known a priori and consistent across all models.

Although centroid size normalization was applied to facilitate registration, this scaling was not retained in the subsequent analysis. Using the established point-to-point correspondences, displacement vectors were computed between the reference model and all remaining models after restoring a consistent physical scale across models, thereby preserving size-related variability [19]. These were assembled into a displacement matrix $\Delta \mathbf{x}$, which served as input for a principal component analysis (PCA), yielding the mean shape and the main modes of variation of the thorax. Based on these components, new thorax models $\mathbf{x}\left({{\boldsymbol{\kappa}}}^{,}\right)$ can be generated as:

1 $$\mathbf{x}\left({{\boldsymbol{\kappa}}}^{,}\right)={\mathbf{x}}_{ref}+\overline{{\boldsymbol{\Delta}}\mathbf{x} }+\sum_{m=1}^{M}{\kappa}_{m}^{,}\sqrt{{\lambda}_{m}}{\phi}_{m}$$

where ${\mathbf{x}}_{ref}$ is the vector containing all points of the reference model, $\overline{\Delta \mathbf{x} }$ denotes the mean displacement, ${\kappa}_{m}^{,}$ is the *m*-th principal component (PC) score, and ${\lambda}_{m}$ and ${\phi}_{m}$ are the corresponding eigenvalues and eigenvectors, respectively, for the *M* retained PCs. The PC scores, ${{\boldsymbol{\upkappa}}}^{,}$, were sampled from a standard normal distribution 𝒩∼(0,1) and constrained to the range [− 3, 3].

The main modes of variation of the developed SSMs were assessed by generating thorax geometries along the most relevant PCs, providing insight into the influence of each component on overall shape. Standard performance metrics reported in the literature, such as generalization, specificity, and compactness, were considered for the validation of the SSM [23]. For brevity, details of the validation procedure are only provided as supplementary material (Online Resource 1).

### Thorax reconstruction from sparse landmarks

Two thorax reconstruction algorithms were developed, each based on a different SSM. One algorithm used the SSM-BL, whereas the other used the SSM-SL. In both cases, reconstruction was formulated as an optimization problem in which the optimal PC scores were estimated by minimizing the discrepancy between model-predicted landmarks and a target set of landmarks. The implementation of the proposed reconstruction algorithms is available in a public GitHub repository to support reproducibility and reuse in future research (https://github.com/PROMOTE-BIO/thorax-ssm-reconstruction). Denoting **x**_target_ as a set of target landmark coordinates, the objective function to be minimized was defined as:

2 $$\mathrm{f}\left({{\boldsymbol{\kappa}}}^{,}\right)=\left[\frac{1}{{N}_{l}}\sum_{i=1}^{{N}_{l}}{\Vert {\mathbf{x}\left({{\boldsymbol{\kappa}}}^{,}\right)}_{i}-{{\mathbf{x}}_{\mathrm{t}\mathrm{a}\mathrm{r}\mathrm{g}\mathrm{e}\mathrm{t}}}_{i}\Vert }^{2}\right]+c\sum_{j=1}^{M}\frac{{\lambda}_{1}}{{\lambda}_{j}}{\kappa}_{j}^{{,}^{2}}$$

where *N*_*l*_ is the number of landmarks (five in this study) and *c* is a regularization weight.

The second term in Eq. (2) acts as a regularization term, encouraging reconstructions that are primarily governed by the dominant modes of variation, associated with larger eigenvalues, while penalizing contributions from higher-order PC scores. In this way, the influence of PC scores associated with smaller eigenvalues is limited, reducing the likelihood of introducing anatomically implausible deformations or overfitting to fine-scale variability.

For tuning the regularization weight *c*, the training set was randomly subdivided into a model-fitting subset (75%) and a validation subset (25%). The value of *c* was selected by minimizing the reconstruction error on the validation subset using SSMs constructed from the model-fitting subset. Once determined, final SSMs were built using the full training data set.

To reflect typical motion analysis data, both reconstruction algorithms were designed to take thorax skin landmark coordinates as input. In the SSM-SL-based approach, these landmarks were used directly as target landmarks in the optimization procedure. In contrast, the SSM-BL-based approach required an intermediate mapping from skin landmarks to bone landmarks, as the SSM-BL only represents bony anatomy. Following the methodology proposed by Marques et al. [5], linear regression models were developed to map the input skin landmark coordinates to corresponding bone landmark coordinates that could be used as target landmarks in the optimization problem. The development of these linear regression models is detailed in Sect. 2.3.1.

#### Skin-to-bone landmark mapping

Fifteen separate linear regression models were developed, each predicting a single response variable, defined as the *x*, *y*, or *z* coordinate of the five thoracic bone landmarks (C7, T8, IJ, PX, and R10), from 26 predictors. The predictors included the *x*, *y*, and *z* coordinates of the five skin landmarks (11 predictors), the Euclidean distances between all pairs of skin landmarks (10 predictors), and subject-specific data, such as sex, age, weight, height, and BMI (5 predictors). As all landmark coordinates were standardized to each individual’s ISB thoracic reference frame, the *z* coordinate of the skin C7 and the *x*, *y*, and *z* coordinates of skin IJ were equal to zero, and were therefore discarded from the predictors.

To ensure robust model development, several linear regression models were evaluated by exploring combinations of regression algorithms, data transformation strategies, and hyperparameter configurations [24]. The regression algorithms included the Ordinary Least Squares (OLS), Least Absolute Shrinkage and Selection Operator (LASSO), Ridge regression, Elastic Net regression, and Partial Least Squares (PLS), all implemented in the *scikit-learn* Python library [25]. Data transformation approaches included no transformation (raw data), z-score normalization, and min–max scaling.

Hyperparameter tuning involved testing regularization strengths of (0.0001,0.001,0.01,0.1,1,10,100) for LASSO and Ridge regression, and all combinations of regularization strengths (0.0001,0.001,0.01,0.1,1) and *l*_*1*_ ratios (0.1,0.3,0.5,0.7,0.9) for the Elastic Net regression. The regularization strength controls the magnitude of penalization applied to the regression coefficients, while the *l*_*1*_ ratio determines the balance between LASSO and Ridge penalization in Elastic Net.

To reduce the risk of underfitting or overfitting, the training set was randomly divided into 10 folds using a shuffle split strategy with a 75%−25% training-validation ratio. Each regression model, defined by a unique combination of algorithm, hyperparameters, and transformation approach, was therefore trained and evaluated ten times for each response variable.

To identify the most relevant predictors for each regression model, stepwise feature selection was performed using Sequential Feature Selection implemented in the *mlxtend* Python library [26]. In total, 1230 linear regression models were evaluated per response variable. The best-performing model was selected as the one minimizing the score

3 $$s=0.5\times \frac{{MAE}^{train}+{MAE}^{val}}{2}+0.5\times \left|{MAE}^{train}-{MAE}^{val}\right|$$

where ${MAE}^{train}$ and ${MAE}^{val}$ are the mean absolute errors (MAE) between the predicted and ground-truth bone landmarks computed over the training and validation subsets, respectively [5].

Once the best combination of regression algorithm, data transformation strategy, and hyperparameters was identified for each response variable, final linear regression models were trained using the full training data set.

### Thorax reconstruction performance

The performance of the proposed reconstruction algorithms was evaluated by applying them to the independent test set. Several error metrics were computed to quantify reconstruction accuracy, and a comparative statistical analysis was conducted.

The accuracy of the skin-to-bone landmark mapping was assessed by computing landmark-to-landmark errors between predicted and ground-truth bone landmarks, using the root-mean-square error (RMSE) and MAE. Thorax reconstruction accuracy was evaluated using the same metrics, computed between the reconstructed thorax shapes and the corresponding ground-truth geometries. These errors were calculated using point-to-point correspondences obtained through the BCPD algorithm across all thorax surface points.

Statistical analyses were performed to compare the distributions of the thorax reconstruction errors obtained with the two reconstruction algorithms (SSM-BL-based and SSM-SL-based) on the test set. Differences between groups were assessed using a two-tailed independent Welch’s t-test. A significance level of 0.05 was adopted for all statistical tests. Effect sizes were calculated using Cohen’s *d* to provide an estimate of the magnitude of observed differences, independently of sample size.

## Results

### SSM modes of variation

**Fig. 2:**
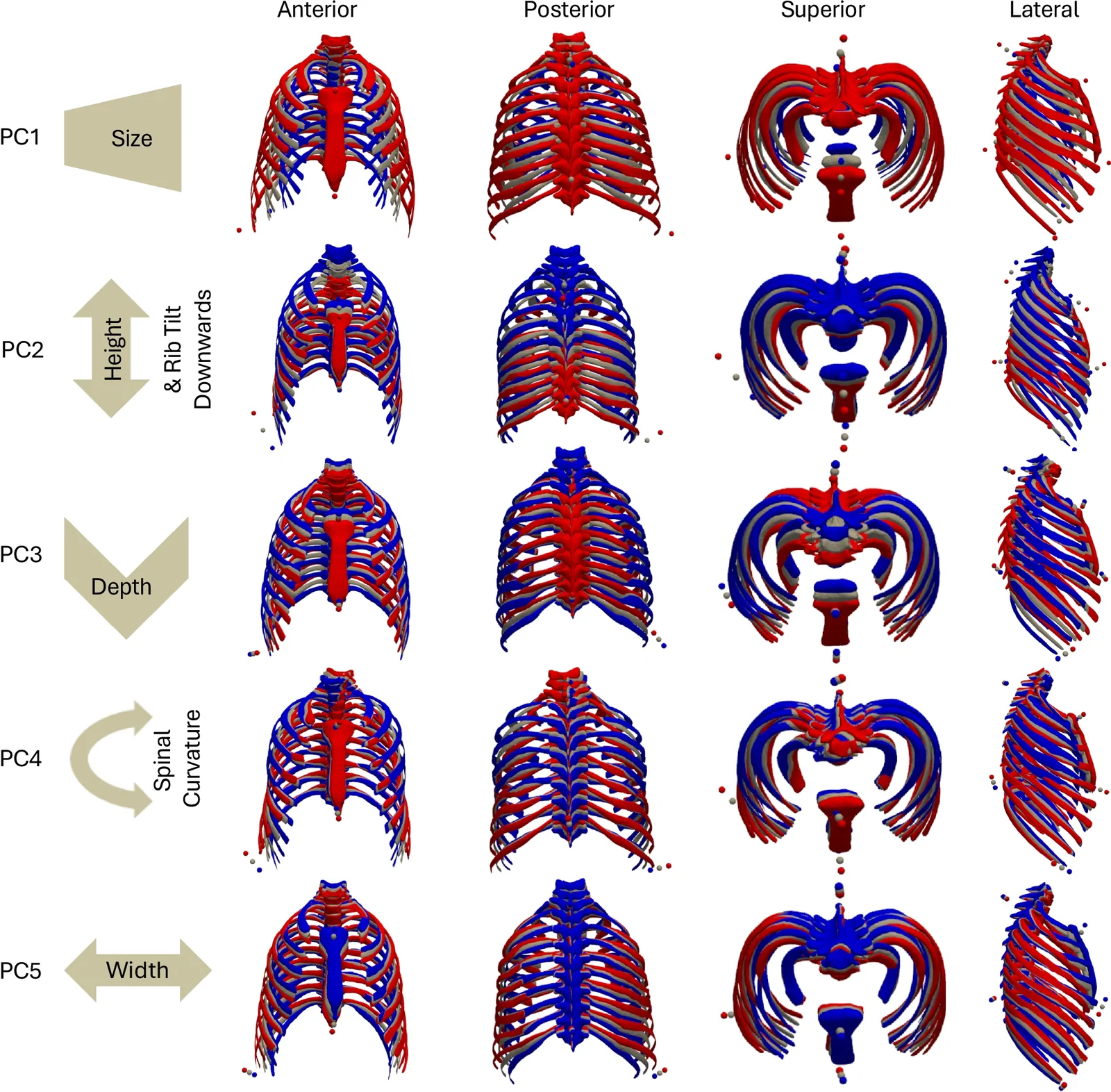
Thorax shape variation for the first five PCs of the SSM-SL. Each PC is shown in four views (anterior, posterior, superior, and lateral), with the variation shown as three superimposed shapes - mean shape (grey) as well as the mean shape with −3 (blue) and +3 (red) standard deviation magnitudes. The skin landmarks are represented as spheres with 5 mm radius, for visualization purposes

The first five modes of variation of the SSM-BL and SSM-SL were analyzed, explaining 68.8% and 72.4% of the cumulative variance, respectively. As these modes were qualitatively similar across both models, only the modes of the SSM-SL are presented for brevity. Figure 2 illustrates the mean thorax shape and the first five modes of variation of the SSM-SL.

The first PC primarily captured overall thorax size, reflecting near-uniform scaling of the thoracic structure. The second PC was mainly associated with variations in thorax height, influencing vertebral and sternal positions, rib spacing, and rib tilt. Negative PC2 scores corresponded to a more barrel-shaped thorax, whereas positive scores corresponded to a flatter thorax with proximal rib downward tilt. The third PC captured variations in thorax depth, affecting sternum and rib positions, and overall thoracic volume. Positive PC3 scores corresponded to deeper ribcages with a straighter upper thoracic spine, while negative scores reflected a flatter thorax. The fourth PC was associated with lateral curvature of the thoracic spine and variations in lower rib width, with positive scores corresponding to wider lower ribs and negative scores to narrower lower ribs. The fifth PC primarily reflected variations in overall thorax width, especially in the lower ribs.

### Skin-to-bone landmark mapping through linear regression

Table 1 summarizes the best-performing multiple linear regression models for mapping skin landmarks to bone landmarks, together with model quality metrics computed on the training set. Model complexity ranged from a single predictor in the simplest model to 15 predictors out of the 26 available in the most complex model. With the exception of the models predicting the *z* coordinate of the C7 landmark and the *y* and *z* coordinates of the IJ landmark, which showed poor to moderate fit, the remaining models demonstrated high explanatory power, with R^2^ values exceeding 0.80.

**Table 1.** Linear regression equations developed to map skin landmarks to bone landmarks. Each equation predicts a single coordinate component (*x*, *y*, or *z*) of a bone landmark (C7, T8, IJ, PX, and R10) from a combination of skin landmark coordinates and anthropometric variables. The symbols A, BMI, H, S, and M represent age (years), body mass index (kg.m^−2^), height (m), sex (0 = female, 1 = male), and body mass (kg), respectively. Landmark coordinates and inter-landmark distances (d) are expressed in millimeters. Model quality is indicated by the coefficient of determination ${R}^{2}$ and the adjust coefficient of determination ${R}_{adj}^{2}$ computed on the training set

**Response Variable**	**Linear Regression Equation**	**Regression algorithm**	**Data transformation**	${{\boldsymbol{R}}}^{2}$	${{\boldsymbol{R}}}_{{\boldsymbol{a}}{\boldsymbol{d}}{\boldsymbol{j}}}^{2}$
${x}_{bone}^{C7}$	$55.423+0.092\times A+0.556\times M-22.250\times H$ $-1.301\times BMI+4.264\times {x}_{skin}^{C7}+1.475\times {y}_{skin}^{T8}+0.668\times {z}_{skin}^{T8}$ $-3.780\times {x}_{skin}^{PX}-0.068\times {z}_{skin}^{PX}-0.975\times {x}_{skin}^{R10}-0.7438\times {y}_{skin}^{R10}$ $+3.635\times {d}_{skin}^{T8-IJ}+1.388\times {d}_{skin}^{T8-R10}-1.909\times {d}_{skin}^{IJ-R10}$	ElasticNet	raw	0.87	0.84
${y}_{bone}^{C7}$	$10.804+1.221\times S+0.014\times A-0.135\times BMI$ $+0.590\times {y}_{skin}^{C7}+0.579\times {x}_{skin}^{T8}+1.014\times {x}_{skin}^{PX}+0.063\times {z}_{skin}^{PX}$ $+0.013\times {x}_{skin}^{R10}+0.808\times {d}_{skin}^{C7-IJ}-0.194\times {d}_{skin}^{T8-PX}$	ElasticNet	z-score	0.98	0.97
${z}_{bone}^{C7}$	$-1.170+8.678\times H+0.143\times BMI+0.045\times {y}_{skin}^{T8}$ $-0.040\times {x}_{skin}^{R10}-0.057\times {z}_{skin}^{R10}-0.030\times {d}_{skin}^{C7-IJ}$	ElasticNet	z-score	0.28	0.20
${x}_{bone}^{T8}$	$-4.452-0.080\times A+0.170\times M+1.025\times {x}_{skin}^{T8}$ $-0.091\times {x}_{skin}^{PX}-0.076\times {x}_{skin}^{R10}+0.065\times {d}_{skin}^{T8-R10}$	Ridge	min-max	0.95	0.95
${y}_{bone}^{T8}$	$-2.490+0.586\times S+0.015\times A-0.039\times BMI$ $+0.563\times {x}_{skin}^{C7}+0.757\times {y}_{skin}^{T8}+0.022\times {x}_{skin}^{R10}-0.223\times {d}_{skin}^{C7-T8}$ $+0.583\times {d}_{skin}^{C7-IJ}-0.047\times {d}_{skin}^{C7-PX}+0.059\times {d}_{skin}^{T8-PX}$ $+0.01572\times {d}_{skin}^{IJ-R10}$	Lasso	z-score	0.99	0.99
${z}_{bone}^{T8}$	$0.497+0.434\times {z}_{skin}^{T8}+0.029\times {y}_{skin}^{PX}-0.464\times {z}_{skin}^{PX}$ $-0.010\times {x}_{skin}^{R10}-0.034\times {d}_{skin}^{C7-IJ}+0.031\times {d}_{skin}^{C7-PX}$	Ridge	raw	0.95	0.94
${x}_{bone}^{IJ}$	$3.955+3.881\times S+0.128\times A-0.239\times {x}_{skin}^{C7}+1.262\times {y}_{skin}^{T8}$ $-0.531\times {x}_{skin}^{PX}-0.451\times {z}_{skin}^{PX}-0.888\times {y}_{skin}^{R10}+1.730\times {d}_{skin}^{C7-T8}$ $-0.509\times {d}_{skin}^{C7-IJ}-1.465\times {d}_{skin}^{C7-R10}+0.712\times {d}_{skin}^{T8-R10}$	ElasticNet	raw	0.87	0.83
${y}_{bone}^{IJ}$	$1.460-0.035\times S-0.026\times A+0.038\times BMI+0.038\times {y}_{skin}^{T8}$ $-0.080\times {x}_{skin}^{PX}-0.026\times {d}_{skin}^{C7-R10}+0.015\times {d}_{skin}^{T8-PX}$ $+0.033\times {d}_{skin}^{IJ-PX}+0.036\times {d}_{skin}^{PX-R10}$	ElasticNet	raw	0.57	0.48
${z}_{bone}^{IJ}$	$-3.796-0.012\times {y}_{skin}^{R10}$	Lasso	z-score	0.06	0.05
${x}_{bone}^{PX}$	$24.489+8.188\times S-0.541\times BMI+0.503\times {x}_{skin}^{C7}$ $+0.035\times {y}_{skin}^{PX}-0.094\times {z}_{skin}^{R10}+0.408\times {d}_{skin}^{T8-PX}$	ElasticNet	z-score	0.85	0.83
${y}_{bone}^{PX}$	$-1.793+0.258\times BMI+0.059\times {y}_{skin}^{T8}+0.835\times {y}_{skin}^{PX}$ $-0.137\times {d}_{skin}^{IJ-PX}$	ElasticNet	raw	0.99	0.99
${z}_{bone}^{PX}$	$-13.980-0.032\times A-0.085\times M+0.150\times {y}_{skin}^{C7}$ $+0.776\times {y}_{skin}^{PX}+1.059\times {z}_{skin}^{PX}-0.1042\times {z}_{skin}^{R10}-0.128\times {d}_{skin}^{C7-T8}$ $+0.380\times {d}_{skin}^{T8-IJ}-0.380\times {d}_{skin}^{T8-PX}+0.184\times {d}_{skin}^{T8-R10}$ $+0.975\times {d}_{skin}^{IJ-PX}-0.262\times {d}_{skin}^{IJ-R10}+0.251\times {d}_{skin}^{PX-R10}$	ElasticNet	z-score	0.94	0.93
${x}_{bone}^{R10}$	$9.257+0.028\times A+0.072\times M+0.915\times {x}_{skin}^{R10}$ $+0.039\times {y}_{skin}^{R10}-0.114\times {z}_{skin}^{R10}-0.083\times {d}_{skin}^{C7-IJ}$ $+0.0857\times {d}_{skin}^{T8-R10}$	ElasticNet	z-score	0.99	0.99
${y}_{bone}^{R10}$	$-4.339-0.086\times {x}_{skin}^{C7}+0.959\times {y}_{skin}^{R10}-0.086\times {d}_{skin}^{T8-IJ}$	ElasticNet	raw	0.99	0.99
${z}_{bone}^{R10}$	$15.738+9.092\times S+0.154\times A-0.317\times M$ $+25.930\times H+4.489\times {y}_{skin}^{C7}-0.733\times {x}_{skin}^{T8}-0.968\times {y}_{skin}^{T8}$ $-1.473\times {y}_{skin}^{PX}-0.360\times {y}_{skin}^{R10}+0.9142\times {z}_{skin}^{R10}-1.698\times {d}_{skin}^{C7-T8}$ $-2.863\times {d}_{skin}^{C7-PX}-0.524\times {d}_{skin}^{C7-R10}+1.270\times {d}_{skin}^{T8-IJ}$ $+0.816\times {d}_{skin}^{IJ-PX}$	Ridge	z-score	0.91	0.88

On the test set, predictive performance varied across landmarks and coordinate components (Table 2). Mean absolute errors ranged from 0.44 ± 0.27 mm for the *y* coordinate of the IJ landmark to 7.77 ± 5.09 mm for the *z* coordinate of the R10 landmark, while RMSE ranged from 0.52 ± 0.61 mm to 9.29 ± 10.24 mm for the same respective coordinates. Excluding four out of the 15 regression models, all models achieved MAE and RMSE values below 5 mm.

**Table 2.** Predictive performance of the linear regression models evaluated on the test set, reported as mean absolute error (MAE) and root-mean-square error (RMSE) in millimeters. Each response variable corresponds to a single coordinate component (*x*, *y*, or *z*) of a predicted bone landmark (C7, T8, IJ, PX, and R10).

**Response Variable**	**MAE**	**RMSE**
${x}_{bone}^{C7}$	5.16 ± 4.65	6.93 ± 8.07
${y}_{bone}^{C7}$	1.75 ± 1.72	2.45 ± 3.21
${z}_{bone}^{C7}$	1.72 ± 1.10	2.04 ± 2.02
${x}_{bone}^{T8}$	3.95 ± 3.49	5.27 ± 6.96
${y}_{bone}^{T8}$	1.25 ± 1.11	1.67 ± 2.03
${z}_{bone}^{T8}$	1.29 ± 1.37	1.88 ± 2.83
${x}_{bone}^{IJ}$	3.41 ± 2.27	4.09 ± 4.32
${y}_{bone}^{IJ}$	0.44 ± 0.27	0.52 ± 0.61
${z}_{bone}^{IJ}$	0.63 ± 0.76	0.99 ± 1.53
${x}_{bone}^{PX}$	5.17 ± 3.75	6.39 ± 6.88
${y}_{bone}^{PX}$	1.33 ± 0.94	1.62 ± 1.69
${z}_{bone}^{PX}$	1.73 ± 1.43	2.24 ± 2.72
${x}_{bone}^{R10}$	1.75 ± 1.72	2.46 ± 3.52
${y}_{bone}^{R10}$	1.84 ± 1.22	2.20 ± 2.29
${z}_{bone}^{R10}$	7.77 ± 5.09	9.29 ± 10.24

### Thorax reconstruction

The reconstruction algorithms were applied to the independent test set considering a regularization weight *c* of 0.1. This parameter was fine-tuned separately for the SSM-BL-based and SSM-SL-based approaches, with both yielding the same optimal value. Table 3 summarizes thorax bone surface reconstruction errors obtained with both algorithms for increasing numbers of PCs, corresponding to cumulative variance levels of 50%, 65%, 75%, 85%, and 90% (see Table 3 caption for the corresponding number of PCs in each model). These variance levels were considered to evaluate the trade-off between reconstruction accuracy and model complexity in the reconstruction process.

**Table 3.** Thorax bone surface reconstruction error metrics obtained on the test set using varying numbers of principal components (PCs) in the SSMs, reported as mean ± standard deviation of the mean absolute error (MAE) and root-mean-square error (RMSE) (mm). Cumulative variance levels of 50%, 65%, 75%, 85%, and 90% correspond to 2, 4, 8, 15, and 21 PCs in the SSM-BL, and to 2, 4, 7, 13, and 20 PCs in the SSM-SL, respectively

	**MAE**		**RMSE**	
**Cumulative variance**	**SSM-BL**	**SSM-SL**	**SSM-BL**	**SSM-SL**
50%	8.35 ± 1.25	8.36 ± 1.70	9.32 ± 1.49	9.28 ± 1.88
65%	8.33 ± 1.48	8.31 ± 1.91	9.29 ± 1.65	9.16 ± 2.08
75%	8.31 ± 1.44	8.30 ± 1.99	9.28 ± 1.60	9.14 ± 2.17
85%	8.28 ± 1.41	8.27 ± 1.94	9.26 ± 1.58	9.12 ± 2.12
90%	8.27 ± 1.41	8.26 ± 1.96	9.25 ± 1.58	9.10 ± 1.12

No statistically significant differences in thorax bone surface reconstruction error were observed between the two reconstruction approaches (Welch’s t-test, *p* > 0.05 for all comparisons). Overall, the MAE ranged from 8.26 ± 1.96 mm to 8.36 ± 1.70 mm, while the RMSE ranged from 9.10 ± 1.12 mm to 9.32 ± 1.49 mm. Increasing the cumulative variance, corresponding to a higher number of PCs, resulted in a modest reduction in reconstruction error, with a maximum improvement of approximately 0.18 mm. In contrast, computational time increased substantially, rising on average from approximately 5 min when using the lowest number of PCs to about 85 min when using the highest number of PCs. Excluding reconstructions based on the lowest number of PCs, the SSM-SL-based approach consistently yielded lower errors than the SSM-BL-based approach; however, these differences were small.

**Fig. 3:**
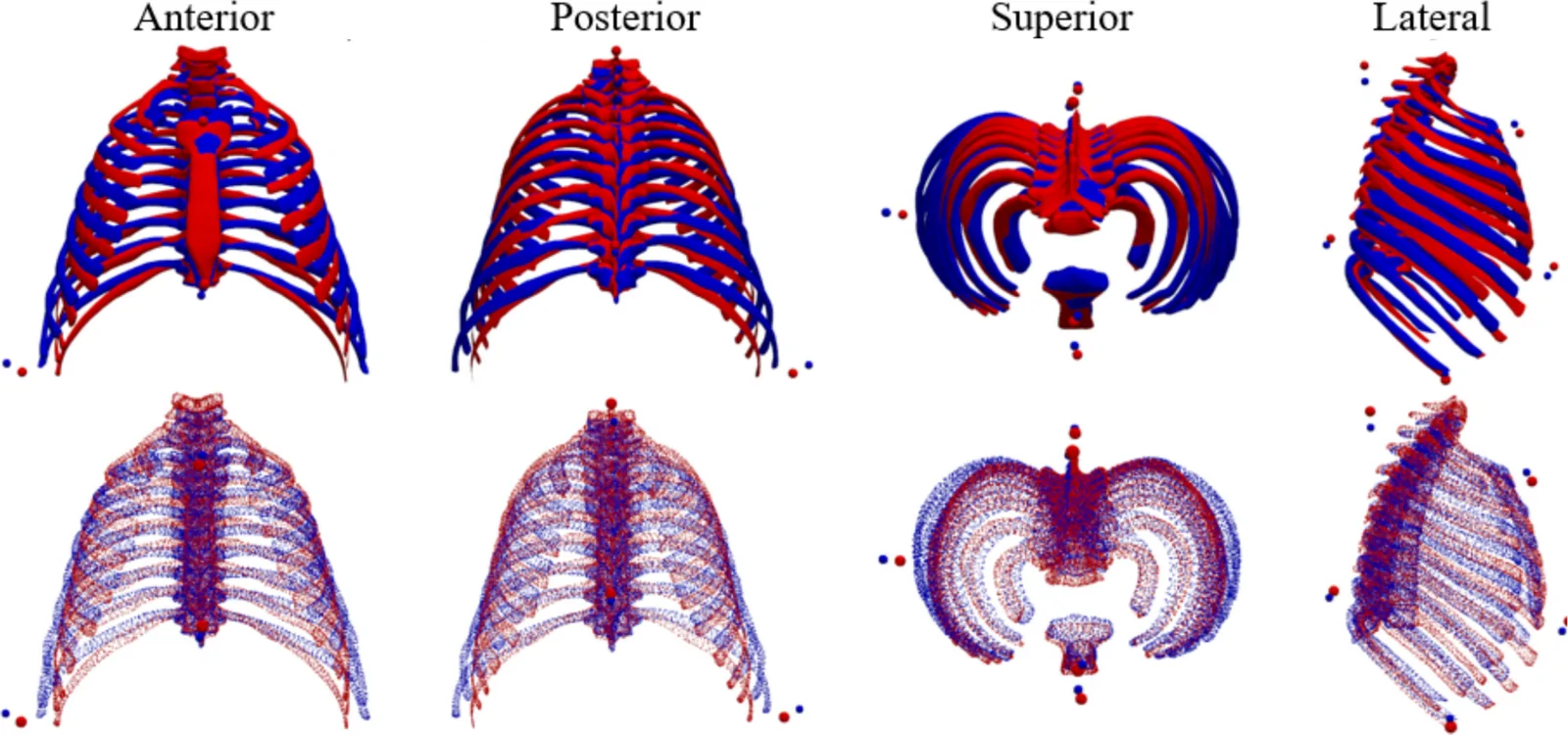
Thorax reconstruction obtained using the SSM-SL-based approach (red) with 20 PCs (corresponding to 90% cumulative variance) for the individual in the test set presenting the minimum bone surface reconstruction error, compared with the corresponding ground-truth thorax model (blue). The top row shows the surface representations, while the bottom row shows the corresponding point clouds. The reconstructed and ground-truth shapes are shown after rigid alignment to illustrate the best possible geometric correspondence

For illustrative purposes, Figure 3 shows the thorax reconstruction obtained using the SSM-SL-based approach with 20 PCs for the individual in the test set presenting the minimum bone surface reconstruction error, alongside the corresponding ground-truth thorax model. While the reconstructed model captures the overall thorax morphology, local discrepancies exist. In particular, the ribcage appears narrower than in the ground-truth model, most noticeably in the lower ribs. In addition, a superior–inferior offset is observed between corresponding ribs of the reconstructed and ground-truth models.

## Discussion

Using statistical shape modeling, the proposed algorithms were able to reconstruct human thorax shapes from a limited set of skin landmarks typically collected in motion analysis studies. However, while the reconstructed models captured the global thorax morphology, local discrepancies were observed, limiting the ability of the reconstructed models to represent subject-specific features and, consequently, restricting the range of applications for which such reconstructions may be suitable. Two reconstruction strategies were evaluated: one in which linear regression models were used to map skin landmarks to bone landmarks that served as targets in an optimization procedure (SSM-BL-based approach), and another in which skin landmarks were embedded directly within the SSM and used as optimization targets (SSM-SL-based approach). The SSM-SL-based approach was intended to avoid the intermediate skin-to-bone mapping step, which was hypothesized to represent a potential source of error. However, no statistically significant differences in reconstruction accuracy were observed between the two approaches.

The reconstruction algorithms relied on two SSMs that differed in the embedding of skin landmarks. For both models, the first five main modes of variation captured the principal thorax variations, including overall size, thorax height, thorax depth, and ribcage width. These modes are consistent with those reported in previous studies [9, 10, 27–29], suggesting that the developed SSMs represent anatomically plausible thorax shape variability.

Overall, the linear regression models developed to map skin landmarks to bone landmarks demonstrated high explanatory power, with most R^2^ values exceeding 0.80. The only regression models with R^2^ values below 0.80 were those predicting the *z* coordinate of the C7 landmark and the *y* and *z* coordinates of the IJ landmark. This behavior is likely related to the limited variance of these coordinates across individuals. When expressed in the ISB thoracic reference frame, these coordinates are expected to be close to zero for all individuals, resulting in a response variable with minimal variance. Under such conditions, the coefficient of determination becomes highly sensitive to small prediction errors, which can lead to low R^2^ values even when absolute prediction errors are small. This interpretation is supported by the predictive performance observed on the test set. Despite the low R^2^ values, both MAE and RMSE for these coordinates remained low, below 1.722 ± 1.096 mm and 2.041 ± 2.019 mm, respectively, indicating good absolute predictive accuracy.

Excluding four out of the 15 regression models, all models achieved mean MAE and RMSE values below 5 mm on the test set. These values appear consistent in magnitude with the predictive errors reported by Marques et al. [5] for similar regression models developed for the scapula, with all 12 models presenting median MAE values below 5 mm. Note that error metrics differ between studies (mean MAE and RMSE values in the present study versus median MAE values in Marques et al. [5]). The poorest performance was observed for the *z* coordinate of the R10 landmark (mean MAE: 7.77 ± 5.09 mm; mean RMSE: 9.29 ± 10.24 mm), corresponding to its medial–lateral component. This behavior may be related to the anatomical location of the R10 landmark, where higher inter-subject variability in skin-to-bone distance was observed in the present dataset, thereby increasing uncertainty in the skin-to-bone relationship.

Application of the reconstruction algorithms to the test set demonstrated their ability to reconstruct thorax shapes from a limited set of skin landmarks, with maximum mean MAE and RMSE of 8.36 ± 1.70 mm and 9.32 ± 1.49 mm, respectively, across the range of cumulative variances considered. Direct comparisons with the literature are challenging due to differences in anatomical scope and error metric definitions. Previous studies on the thorax have often focused on individual thoracic components or have not reported reconstruction errors. Among the limited studies reconstructing thorax geometries as a single structure, Wang et al. [11] used subject-specific variables (age, sex, height, and BMI) as predictors and reported average Euclidean distance errors ranging from 4 to 16 mm across 101 individuals, which are comparable in magnitude to those obtained in the present study.

Although the reconstructed models captured the global thorax morphology, local discrepancies were observed. Even for the test individual with the lowest reconstruction errors, limitations in accurately reproducing rib spacing and local rib geometry were particularly noticeable, likely due to the limited information available for these regions. Only five skin landmarks were used, with just one located on a rib, which constrained the reconstruction of finer anatomical details. An additional contributing factor may be the modeling of the thorax as a single structure rather than as a set of individual thoracic components. As the thorax is not rigid but exhibits flexibility at its articulations, inter-subject differences in posture during image acquisition may have introduced a confounding source of shape variability. In summary, despite reconstruction errors being comparable to those reported in the literature, the accuracy of the proposed algorithms may limit their applicability for tasks requiring precise subject-specific thorax geometry.

The SSM-SL-based approach was designed to eliminate the intermediate skin-to-bone mapping step of the SSM-BL-based approach, motivated by the assumption that this intermediate step could degrade accuracy. Although the SSM-SL-based approach yielded lower reconstruction errors than the SSM-BL-based approach, the differences were not statistically significant. Two factors related to the development of the linear regression models may help explain this outcome. First, the regression models were developed using an extensive evaluation of multiple algorithms, data transformation strategies, and hyperparameter configurations, which likely enhanced their predictive performance. Second, the inclusion of subject-specific variables in the regression models may have provided additional information to characterize the relationship between skin and bone landmarks, whereas the SSM-based reconstruction relied solely on landmark coordinates. Beyond reconstruction accuracy, the SSM-SL-based approach achieved a given level of cumulative variance with fewer PCs than the SSM-BL-based approach (see Table 3), suggesting improved compactness of the model, which may translate into greater computational efficiency. In addition, its implementation is more straightforward, as it directly uses embedded skin landmarks and does not require the development of separate skin-to-bone regression models.

Despite the contributions of this study to the computational modeling of the thorax, several limitations should be acknowledged. Although the sample size is comparable to sample sizes used in previously published studies [5, 7, 9, 10], it remains limited. Increasing the number of individuals can improve the robustness and generalizability of the proposed algorithms. All thorax models were derived from CT scans acquired from cadavers in a supine position, whereas the intended application of the developed algorithms targets individuals in a standing posture. This positional discrepancy may introduce posture-related bias, potentially affecting reconstruction accuracy. In addition, the thorax was modeled as a single structure, which may have limited reconstruction performance by not accounting for variations in the relative positioning of individual thoracic components. Future SSM-based approaches modeling the thorax as a set of interacting components could help improve performance. Finally, only five landmarks were used, reflecting typical configurations used in motion analysis studies. While this choice enhances practical applicability, incorporating additional landmarks may improve subject-specific accuracy.

## Conclusion

The proposed SSM-based algorithms enable the reconstruction of thorax geometry from a limited set of skin landmarks. The reconstructed models captured the global thorax morphology, but local rib characteristics were less accurately represented, particularly in regions with limited landmark information. These findings suggest that, while the proposed approach may be suitable for applications requiring a global description of thorax shape, further methodological developments are needed to improve robustness and subject-specific anatomical detail.

## Supplementary Information

Below is the link to the electronic supplementary material.

Supplementary file1 (DOCX 282 KB)

## Data Availability

The code supporting this study is available at: https://github.com/PROMOTE-BIO/thorax-ssm-reconstruction. The repository includes the implementation of the reconstruction algorithms and example scripts for data processing.
