# Supplementary material for: Thorax shape reconstruction from limited CT-digitized palpable landmarks using statistical shape modeling
Source: Med Biol Eng Comput. 2026 Jul 11;64(8):3115–26. doi: 10.1007/s11517-026-03624-x (PMC13633347; doi:10.1007/s11517-026-03624-x)
Supplement: Supplementary file 1 [file 11517_2026_3624_MOESM1_ESM.docx]

**Thorax Shape Reconstruction from Limited CT-digitized Palpable Landmarks using Statistical Shape Modeling**

**Supplementary Material**

Ricardo Alcobia, João Folgado, Carlos Quental

IDMEC, Instituto Superior Técnico, Universidade de Lisboa, Lisboa, Portugal

**S1. Validation of Statistical Shape Models**

**S1.1 Methods**

Standard performance metrics, including compactness, generalization and specificity [1, 2], were used to validate the statistical shape models (SSMs).

Compactness quantifies how efficiently a model can represent the variability of a set of shapes using a limited number of principal components (PCs). A more compact model explains more variability with fewer PCs, reducing the number of parameters required to accurately reconstruct new shapes.

Generalization quantifies a model’s ability to represent unseen shapes not included in the training set. It was assessed using Leave-One-Out (LOO) validation: for each target shape in the training set, a SSM was constructed from the remaining shapes and used to reconstruct the excluded shape (Equation (1), manuscript). Reconstruction accuracy was quantified as the root-mean-square error (RMSE) between the reconstructed and ground-truth shapes, using the distance from each point on the reconstructed shape to its nearest point on the ground-truth surface across all thorax surface points. The generalization metric was computed as the average RMSE across all LOO iterations [1]. Lower generalization values indicate better performance. A well-constructed SSM is expected to maintain or decrease its generalization error as more PCs are included, demonstrating its ability to represent the population’s variability.

Specificity quantifies how anatomically plausible the shapes generated by a SSM are in comparison with the original training set. It was computed as the average RMSE between 1000 randomly generated shapes and their closest counterparts in the training set. Lower specificity values indicate that the SSM produces shapes closely resembling the population and can generate anatomically coherent geometries.

**S1.2 Results and Discussion**

Figure S1 presents the compactness, generalization, and specificity metrics used to validate the developed SSMs, evaluated up to the number of PCs explaining 98% of the cumulative variance.

The cumulative variance increased gradually. For the SSM-BL and SSM-SL, the first five PCs captured 33.4% and 34.9%, 17.7% and 20.5%, 8.4% and 8.8%, 5.5% and 4.1%, and 3.8% and 4.1% of the training set variance, respectively. These values are consistent with previous studies on human thorax geometries: Bastir et al. [3, 4], Sanchis-Gimeno et al. [5], and Wang et al. [6] reported that the first PC explained approximately 34.5–41.2% of variance, the second PC 11.6–25.1%, the third PC 8.5–15.8%, the fourth PC 6%, and the fifth PC 5%. Cumulative variances of 50%, 65%, 75%, 85%, and 90% were achieved with 2, 4, 8, 15, and 21 PCs for the SSM-BL, and with 2, 4, 7, 13, and 20 PCs for the SSM-SL.

Compactness (%)

Generalization (mm)

Specificity (mm)

**Figure S1**. Validation metrics for the developed SSMs: compactness, generalization, and specificity. SSM-BL results are shown in blue, and SSM-SL results are shown in orange.

The generalization metric decreased as the number of PCs increased, reflecting the greater amount of shape information captured with additional components. For the current dataset, mean generalization values ranged from 2.75 mm to 4.15 mm for the SSM-BL and from 2.65 mm to 4.05 mm for the SSM-SL. Although generalization values for thorax SSMs are not widely reported, the obtained values fall within the ranges reported for other bones. For example, Marques et al. [1] reported mean generalization values ranging between 2.91 mm and 4.63 mm for scapula shapes, while Nolte et al. [7] reported values ranging between 1.00 mm and 3.00 mm.

For specificity, the likelihood of generating anatomically unrealistic geometries increases as the number of PCs grows, resulting in larger deviations from the training set shapes. In this study, mean specificity values ranged from 3.86 mm to 4.79 mm. As with generalization, literature data for thorax SSMs are scarce, so these values were compared with those reported for other bones. For example, Marques et al. [1] reported specificity values ranging between 3.64 and 4.51 mm for scapula shapes, while Nolte et al. [7] reported values ranging between approximately 2.80 mm and 4.20 mm, which are comparable to those obtained here.

Both SSMs demonstrated similar trends in compactness, generalization, and specificity. The SSM-SL, however, performed slightly better, reaching comparable cumulative variance with fewer PCs and showing lower generalization and specificity values. These improvements may be related to the inclusion of skin landmarks, which could have enhanced the alignment and stability of the SSM construction.

Overall, the validation metrics indicate that the developed thorax SSMs are compact, generalize well to unseen shapes, and generate anatomically plausible geometries, with the SSM-SL showing a modest advantage over the SSM-BL.

**REFERENCES**

1.	Marques A, Folgado J, Quental C (2025) Reconstruction of Scapula Bone Shapes from Digitized Skin Landmarks Using Statistical Shape Modeling and Multiple Linear Regression. Annals of Biomedical Engineering 2025 53:9 53:2239–2250. https://doi.org/10.1007/S10439-025-03768-1

2.	Heimann T, Meinzer HP (2009) Statistical shape models for 3D medical image segmentation: A review. Med Image Anal 13:543–563. https://doi.org/10.1016/j.media.2009.05.004

3.	Bastir M, García-Martínez D, Torres-Tamayo N, et al (2020) Rib cage anatomy in Homo erectus suggests a recent evolutionary origin of modern human body shape. Nat Ecol Evol 4:1178–1187. https://doi.org/10.1038/s41559-020-1240-4

4.	Bastir M, García-Martínez D, Williams SA, et al (2017) 3D geometric morphometrics of thorax variation and allometry in Hominoidea. J Hum Evol 113:10–23. https://doi.org/10.1016/J.JHEVOL.2017.08.002

5.	Sanchis-Gimeno JA, Lois-Zlolniski S, María González-Ruiz J, et al (2020) Association between ribs shape and pulmonary function in patients with Osteogenesis Imperfecta. J Adv Res 21:177–185. https://doi.org/10.1016/j.jare.2019.10.007

6.	Wang Y, Cao L, Bai Z, et al (2016) A parametric ribcage geometry model accounting for variations among the adult population. J Biomech 49:2791–2798. https://doi.org/10.1016/J.JBIOMECH.2016.06.020

7.	Nolte D, Ko ST, Bull AMJ, Kedgley AE (2020) Reconstruction of the lower limb bones from digitised anatomical landmarks using statistical shape modelling. Gait Posture 77:269–275. https://doi.org/10.1016/j.gaitpost.2020.02.010
